# COVID-19 Antibody Test/Vaccination Certification: There's an App for That

**DOI:** 10.1109/OJEMB.2020.2999214

**Published:** 2020-06-01

**Authors:** Marc Eisenstadt, Manoharan Ramachandran, Niaz Chowdhury, Allan Third, John Domingue

**Affiliations:** Knowledge Media InstituteThe Open University 5488 MiltonKeynesMK7 6AAU.K.

**Keywords:** Blockchain, COVID-19, coronavirus, decentralized, immunity certification

## Abstract

*Goal:* As the Coronavirus Pandemic of 2019/2020 unfolds, a COVID-19 ‘Immunity Passport’ has been mooted as a way to enable individuals to return back to work. While the quality of antibody testing, the availability of vaccines, and the likelihood of even attaining COVID-19 immunity continue to be researched, we address the issues involved in providing tamper-proof and privacy-preserving certification for test results and vaccinations. *Methods:* We developed a prototype mobile phone app and requisite decentralized server architecture that facilitates instant verification of tamper-proof test results. Personally identifiable information is only stored at the user's discretion, and the app allows the end-user selectively to present only the specific test result with no other personal information revealed. The architecture, designed for scalability, relies upon (a) the 2019 World Wide Web Consortium standard called ‘Verifiable Credentials’, (b) Tim Berners-Lee's decentralized personal data platform ‘Solid’, and (c) a Consortium Ethereum-based blockchain. *Result*s: Our mobile phone app and decentralized server architecture enable the mixture of verifiability and privacy in a manner derived from public/private key pairs and digital signatures, generalized to avoid restrictive ownership of sensitive digital keys and/or data. Benchmark performance tests show it to scale linearly in the worst case, as significant processing is done locally on each app. For the test certificate Holder, Issuer (e.g. healthcare staff, pharmacy) and Verifier (e.g. employer), it is ‘just another app’ which takes only minutes to use. *Conclusions:* The app and decentralized server architecture offer a prototype proof of concept that is readily scalable, applicable generically, and in effect ‘waiting in the wings’ for the biological issues, plus key ethical issues raised in the discussion section, to be resolved.

## I. Introduction

The Coronavirus/COVID-19 pandemic of 2019/2020 is still taking its terrible toll as we write this [1]. Tests for the presence of antibodies *could* offer a way for people who can prove COVID-19 immunity to go back to work [2], [3]. There are, however, challenges concerning the biological premise of ‘immunity’: the strength and longevity of COVID-19 immunity after infection are matters of current debate and research, as are the sensitivity and robustness of the relevant tests [4], [5] and the race to develop a viable vaccine [6], [7].

Given the scale of the pandemic and financial fallout, it is plausible that ‘COVID-19 antibody test / vaccination certification’ (henceforth ‘CAT/VC’), if shown to be robust, will be in great demand. Bearing in mind the legal and ethical implications of such certification, raised in [8], [9] and our Discussion, we feel that for either the current pandemic or a pandemic of the future, the concept of certification has a place, *particularly when the recipient is employed in healthcare or other key sectors.*

But what form should certification take? A signed or stamped letter is the centuries-old default, and straightforward to roll out at scale, as long as there is some point-of-test proof of identity. Our approach is based on the view that for such a sensitive and likely high-value certificate, a paper version is too vulnerable to alteration or forgery (an exception arises in environments that are ‘lower tech’ for socio-economic reasons and we later describe a printed certificate to address this case). A digital certificate makes the most sense, provided that it can be: (i) Privacy-preserving (because as proud as the holder might be of new-found ‘immunity’, personal data can be re-purposed in unpredictable ways [10]), (ii) un-forgeable, (iii) easy to administer, (iv) easily verifiable while still preserving privacy, (v) scalable to millions of users, and (vi) cost-effective.

All of this effort would be wasted without public acceptance, which is increasingly challenging in an era of suspicion about data-collecting apps [11]. Toward this end, we argue not only for the decentralized approach underlying our design and implementation below, but also for its benefits in allowing individuals who have been tested to change their minds and quit the scheme, knowing that even cryptographically encoded data will be ‘orphaned’ (no data pointing to it), rendering it meaningless. Also, in the Supplementary Materials, we emphasize the importance of having strong oversight by an ethics watchdog to ensure best endeavours to avoid unleashing a Pandora's Box of undesirable side-effects.

How best to undertake such a challenge? Modern smartphone apps and several key technologies such as public key cryptosystems and immutable blockchain records offer some tantalizing prospects for the path we envisage, if they can satisfy the above criteria. Below, we look at the methods by which this can be achieved, assuming a scenario involving testing by a known authority (e.g. a healthcare practitioner or pharmacist), as opposed to self-testing at home. This main paper assumes an ‘On-Site Test for Antibodies + Issuance of Digital Certificate Including Photo ID’ in order to explain our approach, and in the Supplementary Materials we describe variations for (a) ‘Issuing Digital Certificate Without Photo ID’, (b) ‘Issuing Paper Certificate’, (c) ‘Off-Site Testing Via External Lab’, and (d) ‘Vaccination + Certification’

## II. Methods

We focus on the design and implementation of a prototype mobile phone app and requisite decentralized server architecture, intended to facilitate verification of tamper-proof test results. Our design involves a novel hybrid architecture based on (a) the 2019 World Wide Web Consortium standard called ‘Verifiable Credentials’, (b) Tim Berners-Lee's decentralized personal data platform ‘Solid’, and (c) a Consortium Ethereum-based blockchain. We work through (d) a plausible use case scenario, then (e) describe the key ‘onboarding’ and certification steps in detail; and (f) provide benchmark tests to anticipate scaling performance.

### A. ‘Verifiable Credentials’ For Digital Certification

**Fig. 1.**
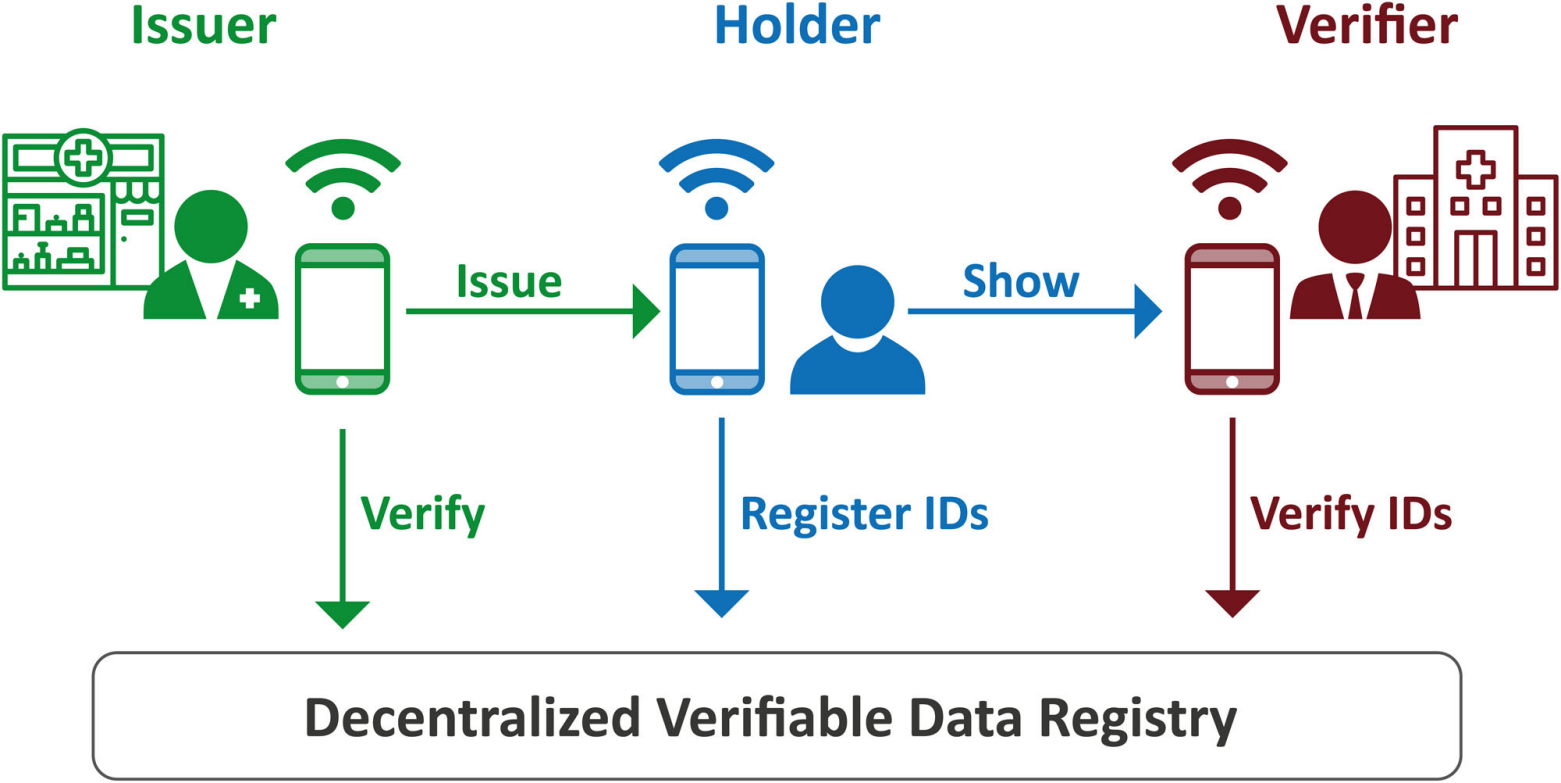
Main roles and workflow in W3C Verifiable Credentials [12], adapted for our COVID-19 Antibody Testing use case.

Verifiable Credentials [12] is a W3C standard that builds upon Public Key Infrastructure (PKI), the public/private key pairs that facilitate digital signatures in widespread use today. The W3C extensions standardize the definitions of document formats to make them machine-readable and communicable, and to generalize PKI, which tends to be costly and highly centralized. The generalization involves a decentralized registry for cryptographic keys, typically residing in a blockchain — this allows every public key to have its own unique address, known as a Decentralized Identifier (DID). The key roles and transactions, adapted for our specific use case, are illustrated in Fig. 1.

The ‘Issuer’, in our case a trusted pharmacy or the UK National Health Service (NHS), can issue credentials such as blood test results and vaccination certificates. ‘Holders’ (typically citizen end-users) can store them in their own preferred way, for example in digital wallets that are part of a mobile phone app. ‘Verifiers’, such as employers, or establishments seeking proof of some attribute, can ask the Holder to present such proof concerning these credentials. Verifiers also check digital signatures against what is known as a ‘verifiable (decentralized) data registry’: this is the blockchain where the DIDs mentioned above reside.

### B. ‘Solid’: Decentralized Personal Data

We pointed out in [13] that the over-centralization of data, particularly its consolidation into ‘silos’ by brand-name IT services and social network providers, is of increasing concern. Decentralization is an ideal starting point for storing sensitive data, including medical, financial, and other personal data — but only if security and privacy are significantly better than what can be offered by traditional centralized systems.

We identified a promising approach to widespread deployment, known as Solid, initiated by Sir Tim Berners-Lee [14], [15]. Solid aims to decentralize the Web by transferring control of data from a central authority to users, thereby allowing users to retain complete ownership of their data, which they store in what are called ‘Solid Pods’ — analogous to a personal web server that is hosted either locally on a mobile phone, or hosted with a cloud provider of the individual's choice, or both. The key distinction from centralized approaches is that even in the provider-hosted case, the provider's access to the data is limited by the user's preferences.

**Fig. 2.**
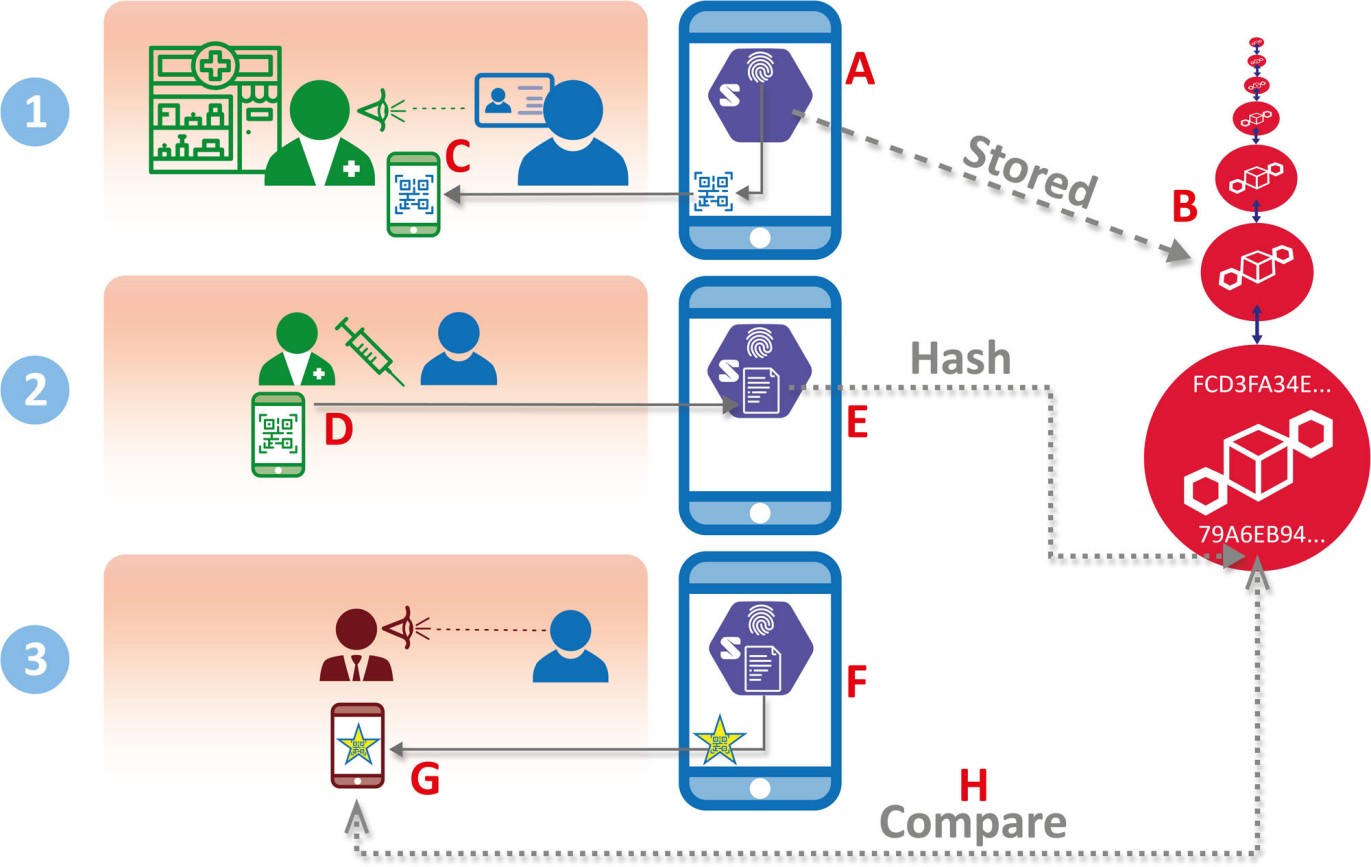
Steps in testing, certification, and verification, showing a Solid Pod (hexagon) hosted on the Holder's mobile phone (labels A, E, F), with minimal hash storage for verification. The circles at B depict replicated blockchain nodes on multiple servers, receding into the distance.

In [16] we proposed an approach combining Solid Pods and distributed ledgers, of the type familiar to the blockchain community, to facilitate the complete decentralization of data. The key ingredients of this combination are illustrated in Fig. 2, which also provides an overview of the main test/certify/verify life cycle. Our methods give users total control over their data while maintaining the integrity of the stored information through blockchain-based verification.

As in Fig. 1, the ‘Holder’ is the primary individual who is self-motivated to obtain the certificate of COVID-19 antibody test results in order to be admitted to a workplace or other location. Holders own, manage, and control their own Solid Pods (shown as hexagons in the Holder's mobile phone in Fig. 2 at A, E, and F), which contain their personal data. In Fig. 2, our Holder's Solid Pod contains a elements of a physical ID such as a driving license (‘thumbprint’ icon at A) and the Holder's signed and countersigned certificate of COVID-19 antibody test results — represented in Fig. 2 as a document in which is embedded a special QR code (F). The Holder is free to store the Solid Pod data on his/her mobile phone, on a personal favorite cloud provider, or both (we only show the mobile phone version for simplicity). At any time, Holders can move or delete data, as it remains under their ownership. One-way encoded ‘hashes’ of the data (only a few bytes in size) are held, as shown by the dashed arrows in Fig. 2 (E and H), on a blockchain to support independent verification.

### C. Consortium Blockchain

In our design, we use a ‘Consortium blockchain’, shown in Fig. 2(B) as circles (depicting multiple replicated blockchain nodes receding into the distance): this is not a fully public blockchain like Ethereum or Bitcoin, but rather a blockchain shared specifically by a *Consortium* of known providers who have signed up to the Ethics Guidelines we describe in the Discussion Section. The Open University-led Consortium blockchain is a private Ethereum network known as OpenEthereum (formerly Parity Ethereum) [17], [18] which uses a ‘Proof of Authority’ consensus mechanism [19] wherein several nodes can be in the mutually-agreed privileged position of being allowed to confirm transactions. As we go to press, our Consortium blockchain comprises nodes run by The Open University, BT, Condatis, Inrupt, and the Chiba Institute of Technology near Tokyo, with expansion planned as our prototype implementation is scaled up via other large-company partnerships now under discussion. This approach contrasts with that of Bitcoin and other early blockchains which use the slow and ecologically unfriendly Proof of Work, wherein massive computing power enables nodes to have a better chance of confirming transactions. The Consortium approach gives us the kind of distributed scalability that increases security, but without the widespread public availability that may serve as a disincentive for individuals to participate.

### D. Use Case Scenario

In our scenario, the Issuer (Pharmacy) needs to authenticate that the Holder is who they say they are, and thus requests that the Holder display (a) a physical ID, such as a Driving License or a Passport, and (b) a QR code which is scanned by the Issuer using the Issuer's mobile phone app, both of which are shown in Fig. 2 (C). At this point the Issuer taps to accept the ID, and the Holder's photo is ‘burned’ into the upcoming steps so that at the final step of verification there will be no need to display the same physical ID. The next steps are as follows:

2) 2) The blood test is performed, and the certificate with results is issued as soon as the results are available (off-site lab tests are dealt with in the Supplementary Materials). The Issuer (first scanning a printed QR code if preferred) generates a digitally-signed test result as a new QR code (labelled D in Fig. 2) for transmission to the Holder, thereby providing a Verifiable Credential which is digitally signed by both the Issuer and the Holder, and stored on the Holder's Solid Pod (Fig. 2, D and E). At label E we also see that a hash of the Verifiable Credential is stored on the Consortium blockchain to facilitate verification at step 3.
3) 3) The Holder can now present a provably valid certificate to the Verifier. To avoid someone else impersonating the Holder, the Holder's ID photo was already ‘burned’ into the digital certificate at Step 1, so the Holder needs to present only the QR code (F and G in Fig. 2)

At H in Fig. 2 we see that the Verifier's app automatically verifies both digital signatures and the certificate against the hash stored on the Consortium blockchain, and confirms acceptance of the COVID-19 Antibody Test Certificate. The certificate stores quantitative test results, such as antibody type (e.g. ‘IgG’) and level, so it is up to the Verifier's own contextually guided procedures to decide whether to admit the Holder, for example, to work.

### E. Primary design (Onboarding and Certification)

Below we separately describe the details for (i) ‘Onboarding’ for Issuers, Holders and Verifiers, and (ii) how Certification works behind the scenes. The companion step of (iii) Verification is conceptually similar, and thus provided separately in the Supplementary Materials, as are the more straightforward descriptions of the server and mobile app functional architectures.

#### 1) Onboarding

There are three entities involved in the operations: Issuers, Holders and Verifiers. The onboarding process lets all of them install and configure the app. The configuration process for each of them is distinct and requires specific documentation.

**Fig. 3.**
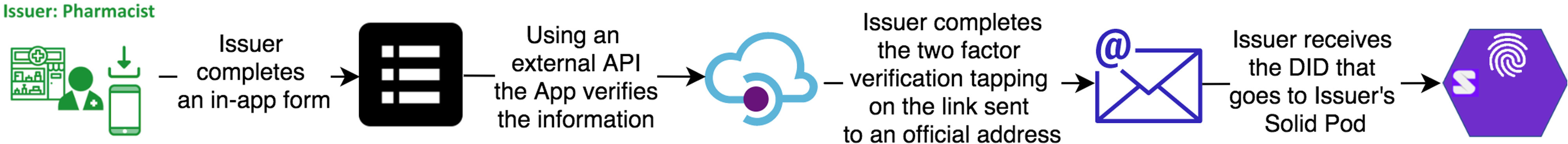
Issuer onboarding timeline details.

**Issuers:** The onboarding of a potential Issuer (Fig. 3) begins with the person downloading and installing the app. The app then instructs the Issuer to complete an in-app form. Because the Issuer has the ability to test, validate and issue certificates to individuals, the app employs *two factor authentication* for all potential Issuers. We anticipate using the API provided by the General Pharmaceutical Council, or an equivalent, to cross-check the registration and the branch information of the likely Issuer (this is simulated in our prototype — discussions about API access are underway), followed by email verification. The former requires the person to input appropriate information into the form, while the latter asks the potential Issuer to provide a valid official email address at the company's registered domain name. The app sends a special link to that Issuer's email address to complete the registration. Data provided by the potential Issuers resides on each Issuer's Solid Pod.

**Fig. 4.**
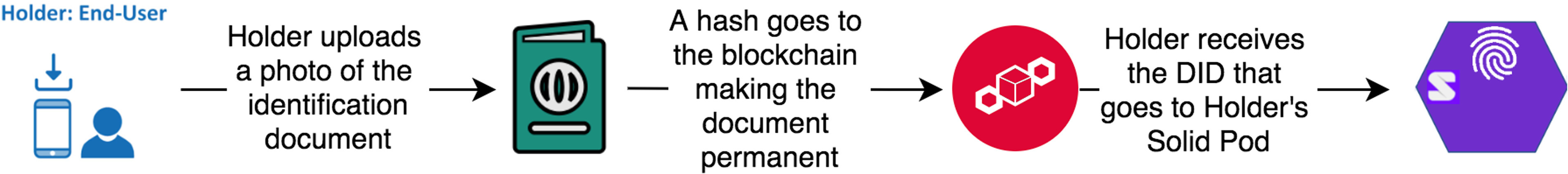
Holder onboarding timeline details.

**Holders:** The process of onboarding a Holder (Fig. 4) involves adding an identification document such as a driving license or passport. The document number is used to generate the Decentralized ID (DID) that acts as the anchor for the Holder. A potential Holder first downloads and installs the app followed by adding a photo of the identification document. This document resides in the Holder's Solid Pod. This photo document is deemed permanent (but remains on their personal Solid Pod) and once submitted, cannot be changed again. The app then provides the Holder with the DID, leaving the owner of the account ready for testing and certification.

**Fig. 5.**
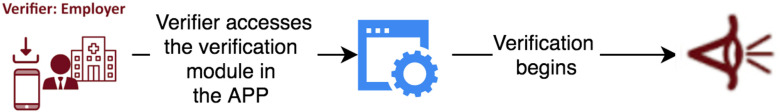
Verifier onboarding timeline details.

**Verifiers:** Of the three main roles, the process of onboarding Verifiers is the most straightforward. Anyone willing to act as a Verifier can download the app and start verifying. There is no need to create an account for verifying a Holder's certificate. As the Verifier submits no data, the steps of the Verifier onboarding timeline (Fig. 5) do not involve Solid Pods.

#### 2) Certification

**Fig. 6.**
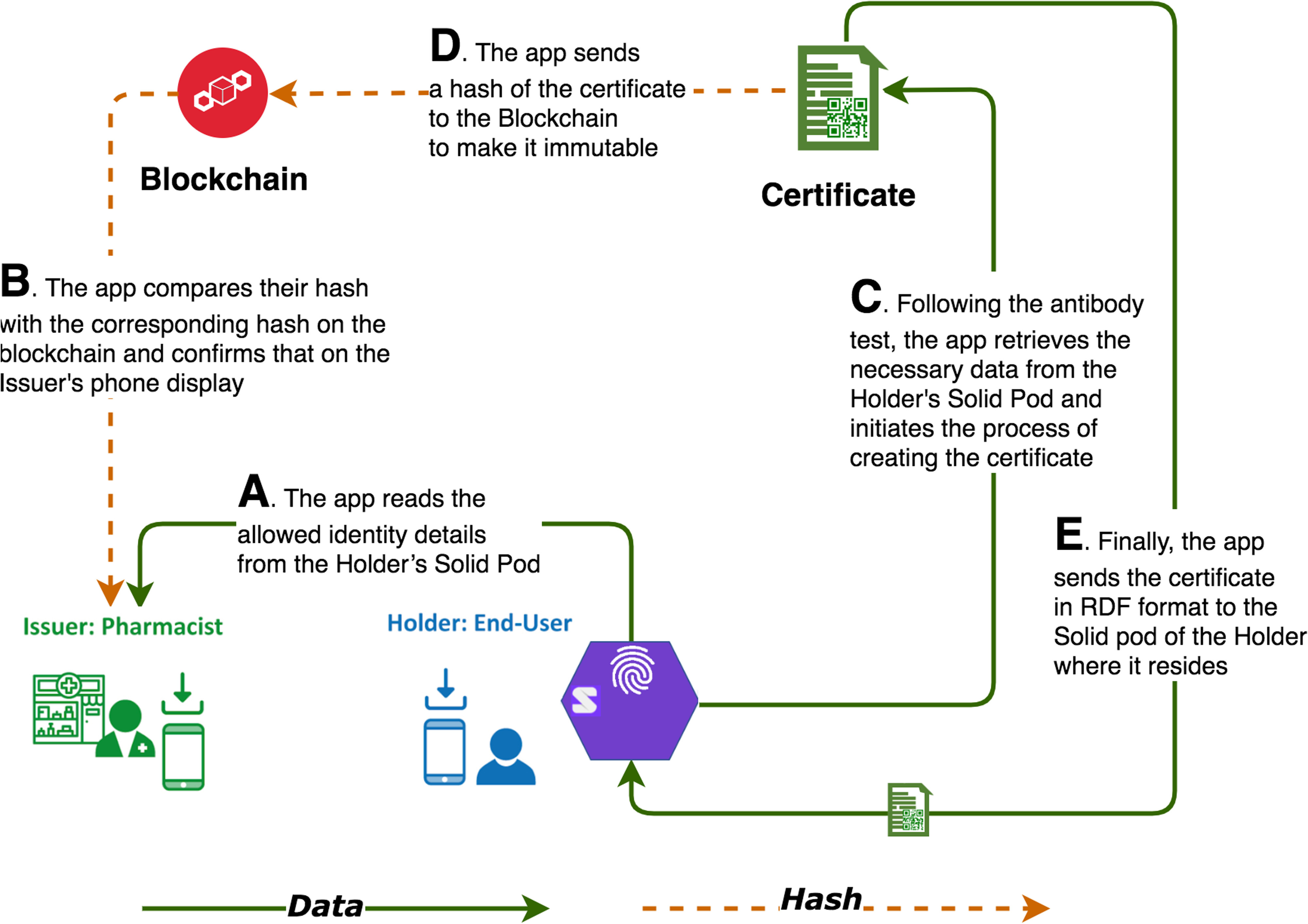
Certification: main dataflows.

The certification process requires a Holder to visit an Issuer with the exact document used for identification at the time of onboarding. At this point the Issuer matches this document with the copy stored in the Holder's Solid Pod, viewing it on the app and tapping to accept the ID. The Holder's photo is ‘burned’ into the upcoming steps so that at the final step of verification, there will be no need to display the same physical ID. In Fig. 6, we see the ‘behind the scenes’ view of certification, including the Holder's Solid Pod with the ID.

The app is designed to work in a completely decentralized environment. Its functionalities run across the Issuer's, Holder's, and Verifier's phones as well as on the hosting servers, but does not have access to any data from a central database. Every time the app needs to execute an operation, it reads the data from a particular user's Solid Pod (and only with the user's permission). In Fig. 6, at (A) we see that the app reads the allowed identity details from the Holder's Solid Pod, and at (B) compares their hash with the corresponding hash on the blockchain and confirms this on the Issuer's phone display.

Once the identity is confirmed, via physical document checks and Verifiable Credentials demonstrating ownership of the relevant DIDs, the Issuer conducts the antibody test and initiates the process of generating a certificate at (C). A certificate is a set of data in W3C RDF (Resource Description Framework) format [20] containing the test results and a Verifiable Credential for the just-tested Holder. While the hash of the certificate goes onto the blockchain at (D), the original document resides in the Solid Pod (E). It is notable that neither the blockchain nor a third-party centralized server stores the personal data of the Holder.

The Holder has the option of keeping a copy of the certificate in a cloud server of his or her choice. In the event of losing the phone, the Holder can retrieve the data from the cloud and restore the certificate in the regenerated local Solid Pod of the replacement phone. This certificate is visible on the Holder's app in the form of a QR code, giving an easy-to-scan option for Verifiers.

#### 3) Verification

The innards of Verification are conceptually similar to what we have just shown for Certification and are thus provided separately in the Supplementary Materials.

### F. Benchmark Testing

To anticipate scalability, we benchmarked three operations (Issuing, Verifying, and Uploading) against a baseline ping that simply echoed a response following a request.

For both Issuing and Verifying we used two variants, to assess the difference between generating hashes (a) locally within the mobile phone app and (b) externally on a server before adding to the blockchain. The Uploading times are purely the times for uploading a certificate to a Solid Pod stored in the cloud, in case that is the Holder's preference.

## III. Results

### A. COVID-19 Antibody Test Certification: App Characteristics

Our ‘COVID-19 antibody test certification’ (CAT/VC) app builds upon the Verifiable Credentials and Solid frameworks described in Section II, plus our own expertise developed over the past 5 years in the area of blockchain-based certification [21], [22]. The result combines the following characteristics:

• Wholly resident on the end-user's smartphone, yet usable as a paper-only certificate in appropriate socio-economic contexts, as described in the Supplementary Materials.
• One-tap scan, display, and verification of antibody test results, which are owned by the user.
• The app only reveals verifiable CAT/VC results without revealing any personally sensitive information, at the discretion of the user.
• The details of Verifiable Credentials, Solid Pods, and Ethereum blockchain are hidden: from the user's point of view, it is ‘just another app’.

### B. Performance Benchmarking Results

**Fig. 7.**
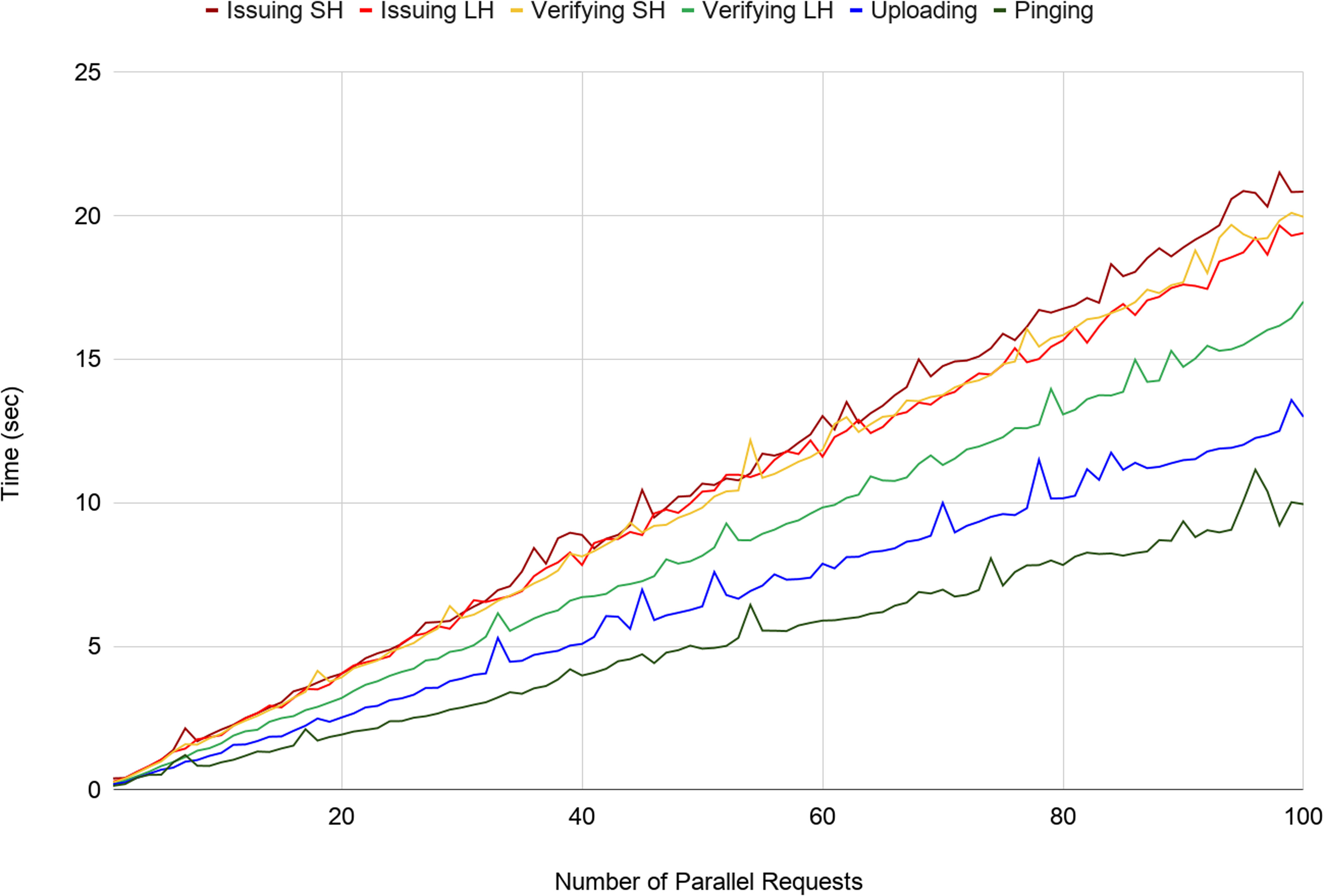
Time to issue up to 100 parallel requests for ‘Issuing’ (SH=Server Hash and LH=Local Hash), ‘Verifying’ (SH=Server Hash and LH=Local Hash) and Uploading of Solid Pod data vs baseline standard ‘Ping’.

Fig. 7 shows the time to completion in seconds (Y axis) of all six operations where we sent between 1 and 100 simultaneous requests (X axis): the fastest (baseline) ping is the lowest line. Uploading is the second least expensive operation, while Verifying and Issuing are the two most expensive operations of our app. The relative difference in time between operations involving locally generated hash (LH) and server-generated hash (SH) is modest for Issuing (6.9% difference between ‘Issuing SH’ and ‘Issuing LH’), but more twice that for Verifying (17.1% difference between ‘Verifying SH and ‘Verifying LH’). This behavior is understandable, as Issuing requires writing on the blockchain through transactions (i.e. the method that allows adding an entry to the distributed ledger) while Verifying involves only a look up at a particular ledger entry.

Linear growth for all operations indicates that our architecture is capable of handling scale-up without surprise: there is simply no inter-node or inter-app communication or interaction overhead, so by improving the configuration of the common infrastructures in the architecture, such as any Solid cloud server, blockchain node, or any other intermediate element, the architecture can serve more parallel requests, i.e. reduce the response time. Implications and additional results are discussed below and in the Supplementary Materials.

## IV. Discussion

### A. Deployment, Integration, and Scale

Our focus is on trusted certification, and for this reason we remain committed to deployments involving nationally approved locations such as pharmacies or UK National Health Service surgeries rather than home testing (off-site lab tests are described in the Supplementary Materials). With our approach, deploying the decentralized servers requires another dozen or so Consortium members in addition to the five already engaged, plus about two days of training, which can be handled in parallel for all Consortium members via webinars, as we already do in our current work with blockchain-based educational certification [23]. The mobile phone app itself requires just a download and less than 30 minutes of training for Issuers, and even less for Verifiers and Holders—we anticipate developing a video tutorial for all scenarios. More significantly is the ‘buy-in’ i.e. acceptance by certified pharmacies and, in the UK, the National Health Service, and integration with existing work practice, ethics guideline approval, and agreement about what, if any, data needs to be stored centrally (no central storage is required at all by our approach). For a full-scale rollout, it would be necessary to further stress-test our prototype along the lines we have already started as described in the preceding Sections.

The technology itself is inherently scalable as our Results section shows: transactions on the Consortium blockchain typically take under 5 *seconds* to be confirmed after entry by the Issuer, after which other steps such as verification are subjectively instantaneous. This scales well, as the architecture is inherently distributed across servers (blockchain nodes) and mobile phone apps. Moreover, we have shown worst case results, covering the case when (a) all Solid servers are hosted on the same machine, (b) all blockchain transactions are being sent to the same specific node, and (c) all users are acting simultaneously. In the best case scenario, all simultaneous users would connect to their own Solid servers, and any simultaneous blockchain transactions would each involve different blockchain nodes, so performance overhead would be constant for each additional user. Realistically, i.e. in between these cases, there would be ample numbers of Solid servers, many dozens to hundreds of blockchain nodes, and natural spacing between transactions, and thus performance overhead for each additional user would be minimal.

Collaborative possibilities for rollout and integration are promising, as new initiatives in this niche are rapidly emerging [24].

### B. Beyond Antibody Test Certification

Our scenario highlights antibody testing, but the technology is identical for vaccination certification, as we describe in the Supplementary Materials — this may prove even more popular once vaccines have been suitably tested and approved [6], [7]. The app and decentralized server architecture are readily scalable and applicable generically. For example,

• People could demonstrate that they are eligible to use different methods of transport or to visit public places such as libraries, theaters, or holiday destinations.
• Utility/building/repair staff seeking access to a place of residence, even in ‘normal’ healthy times, could ‘prove their roles’.
• More generally, the entire area of ‘Decentralized Verifiable Personal Health Records’, as described in [25], particularly if augmented by the W3C Verifiable Credentials standard [12], can benefit from the approach described herein.

### C. Ethics

New technologies bring new challenges for society. Commentators have argued (e.g. in [8], [9], [26]), that certification of the type we have envisaged, even when totally private and tamper-proof, would entail multiple risks, notably: (a) disenfranchising the poor and others who do not have access to the technology or the tests, or have access but ‘fail’ the test, and (b) becoming a stepping-stone for future governments to deploy the same concept either to enable or to enforce discrimination based on immunity and other arbitrary conditions. To avoid this technology becoming ‘weaponized’ for discriminatory purposes, we advocate several measures including optional rather than mandatory use, adherence with UK NHS Information Governance guidelines [27], [28] and oversight by an Ethics Committee. This issue is analyzed in detail in the Supplementary Materials.

## V. Conclusion

The perceived need for a COVID-19 Antibody Test / Vaccination Certificate, if shown to be biologically robust and to conform to proposed ethical guidelines, has motivated us to develop a mobile phone app based around Verifiable Credentials, distributed storage of cryptographic public/key pairs, and the decentralized verification of data with confidentiality. This has enabled us to provide a facility that is ‘just another app’ from the viewpoint of the end-user, healthcare professionals, employers and other relevant authorities — thereby providing a tamper-proof record owned entirely by the end-user, and allowing the end-user selectively to reveal solely the proof of test results without surrendering other personal information (e.g. age, address, blood type, other discovered antibodies or immune deficiencies or other inadvertent revelations in the data set, for which certificate Holders may have no idea how this information might be used by someone else in the future), and requiring only mobile phone app downloads from everyone in the loop. This app and its secure digital certificate thus become a powerful adjunct/enhancement to traditional paper-based certification from the NHS or Pharmaceutical testing authorities — and without the need for the costly installation of special ‘e-ticket reader’ hardware: the same mobile phone app is sufficient for the task at hand, regardless of which of the three roles is involved. Many other uses of secure and private certification via mobile phone app and decentralized servers are additionally made possible, and our infrastructure can be embedded into any other app or web portal through APIs.
