# Supplementary material for: COVID-19 Antibody Test/Vaccination Certification: There's an App for That
Source: IEEE Open J Eng Med Biol. 2020 Jun 1;1:148–55. doi: 10.1109/OJEMB.2020.2999214 (PMC8043425; doi:10.1109/OJEMB.2020.2999214)
Supplement: Supplementary Materials [file supp1-2999214.pdf]

Supplementary Materials

COVID-19 Antibody Test / Vaccination Certification

There's an app for that

Marc Eisenstadt, Manoharan Ramachandran, Niaz Chowdhury, Allan Third, John Domingue*

I. INTRODUCTION

IN this supplementary paper we first discuss the underlying premise of immunity, and then, in the Methods section, provide details of how we deal with privacy and the design of key aspects of onboarding, and verification. In the Results section, we describe additional considerations involved in the system's performance. In the Discussion section, we expand on rollout and the complex ethical issues raised by the research.

The premise of immunity: Throughout most of the COVID-19 pandemic, the World Health Organisation (WHO) has advocated a 'test-isolate-trace' approach [1]. In parallel, there has been a worldwide cooperative effort to develop a vaccine [2] and to develop numerous serological tests for the presence of antibodies [3]. If immunity is strongly implied by the outcomes of these latter tests, then individuals could be allowed to get back to work, particularly in healthcare and other key areas [4], [5]. The WHO initially warned that the very premise of COVID-19 immunity was itself uncertain [6]. Yet the fast pace of research is already showing promising signs that early testing was flawed, the presence of antibodies in recovered individuals has been confirmed, and re-infection now seems increasingly unlikely [7], [8]. True, some immunologists have argued that COVID-19 immunity could be very weak, because 'reinfection is an issue with the four seasonal coronaviruses that cause about 10% to 30% of common colds' [9]. But others in that same discussion argue that immunity could be valid for 'a year or two', a view shared by Male, who with Golding and Bootman has written a clear exposition on the life-cycle of infection, antibody detection, and likely immunity to COVID-19 [10]. A related challenge is the *quality* of the testing: test *sensitivity* (% positive detection for the right antibodies, so high sensitivity means few false positives) and *specificity* (% negatives correctly detected, so high specificity means few false negatives) are undergoing great scrutiny even as we write this [11], and are naturally a matter of concern, because they must be sufficiently high to make the approach worthwhile. In the meantime, our research aims to find an approach to achieve highly robust certification, so that it is ready to deploy as-and-when the ongoing biological research satisfies the necessary quality criteria.

II. METHODS

A. The design of robust privacy

Several important guidelines concerning privacy were set out by the Sovrin Foundation, a nonprofit organisation with over 70 corporate partners including IBM, Cisco and others, which has the aim of 'driving greater interoperability and a new trust model for securely sharing private information' [12]. We adopt a variation of the three principles set out in the Sovrin.org White Paper [13], modifying their item 2 as shown below.

1) Pairwise-unique DIDs and public keys

As Sovrin.org explains, *'Imagine that when you open a new account with an online merchant, instead of giving them a credit card number or phone number, you gave them a DID created just for them. They could still use this DID to contact you about your order, or to charge you a monthly subscription, but not for anything else. If* […] *your DID were compromised in any way, you would just cancel it and give them a new one—without affecting any other relationship.* [consequently…] *a pairwise-pseudonymous DID is not worth stealing.'* [13]

2) Minimum and Encoded Data Storage / User's Choice

According to [13], *no* private data should be stored on the ledger, even in hashed form, to make it future-attack-proof. Sovrin accepts, as do we, the need for pseudonymous identifiers (DIDs), pseudonymous public keys, and agent addresses (e.g. the mobile phone app endpoints) to be stored in a decentralized ledger, but in addition we offer the user a *choice* regarding whether and where to host personal information (mobile phone, favorite cloud provider, or both), plus the barest minimum for verification purposes, namely *hashes* (irreversible encodings) of private data. This has the following benefits:

- Serves as a user-storage 'vault' for later recovery in case of loss.
- This 'vault' (i.e. the Solid Pod) can reside on the user's phone, or on a favorite cloud provider, or both — it is always the user's choice.
- To facilitate later independent verification, it uses a blockchain with distributed nodes run by a Consortium of trusted providers so that there is neither a single point of failure nor a single 'owner' even of the hash of the certificate.
- Even so, it only stores a hash on the Consortium blockchain — a non-reversible but provably correct

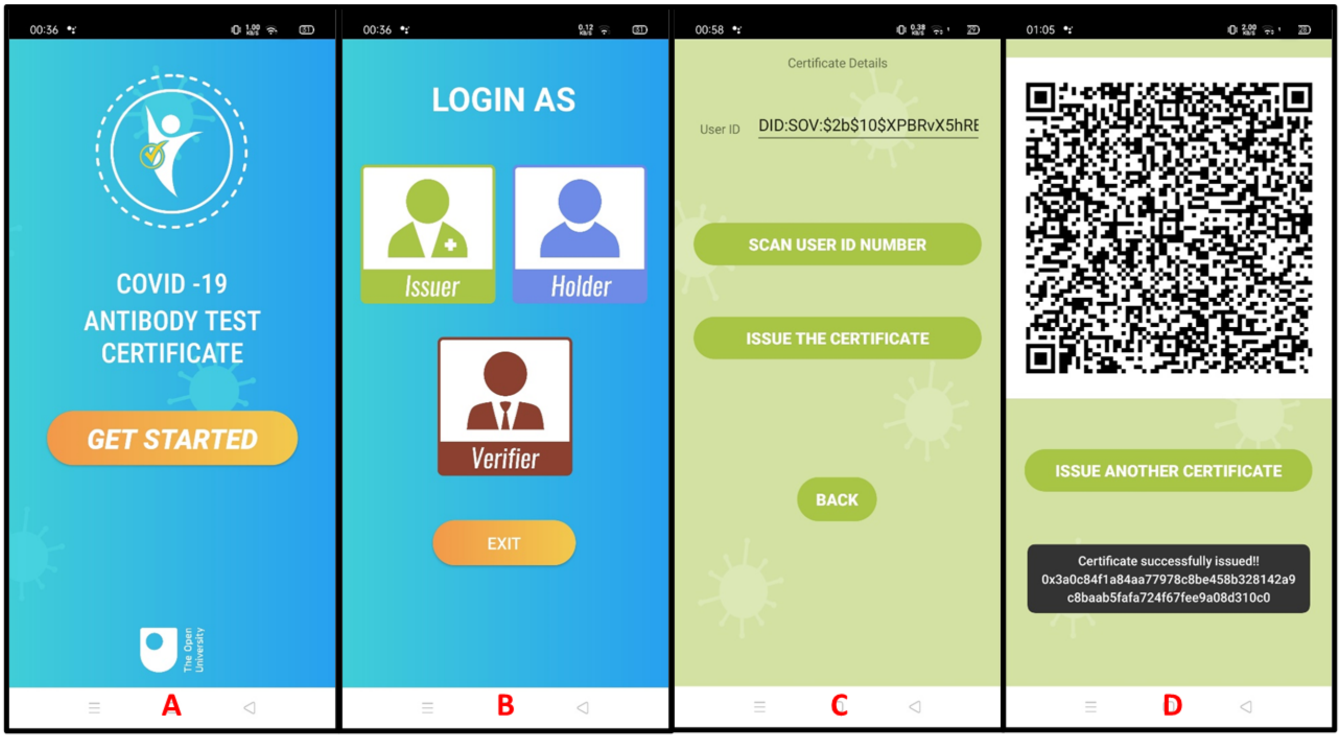

Fig. S2. Representative screen shots of the running mobile app showing (A) home screen, (B) multiple routes for login for the three main roles, just about to tap on 'Issuer', (C) about to issue the certificate having already scanned the user's ID number, displayed at the top, (D) certificate QR code, ready to be scanned by the Holder's mobile phone app.

[illegible]

encoding of the certificate rather than the certificate itself.

This is a powerful privacy-preserving and tamper-proof approach that we call Minimum and Encoded Data Storage / User's Choice. Verborgh [14] has a deeper discussion of the nature and importance of these types of emerging paradigm shifts.

3) Selective disclosure

It is essential that users (certificate Holders) should only have to reveal just the portions of their own personally-held private data that are relevant to specific transactions (e.g. proving that you are 18 years of age or older, in order to make certain purchases or access certain locations, but without revealing your actual age or date of birth). This is made possible by the technology known as *cryptographic zero knowledge proofs* [15–17], so named because they provide, to the Verifier who wishes to know, proof of something specific (such as 'Age ≥ 18'), but with the Verifier having no knowledge of any other details, in this case actual age or date of birth. The 'secret sauce' of zero knowledge proofs, as illustrated in [16], [17], is that a mathematical function works through a proof of some fact (such as age being greater than or equal to X, or the existence of a certain credential), in such a way that the actual steps involved in executing the proof only reach a positive outcome if the fact is true (for example, the positive outcome may require a certain number of steps to execute): so the proof is valid, but still only indirect (e.g. counting the steps executed) without touching the raw data [15], [16].

B. Verification and implementation details

This section describes the operations that underpin the functioning of *verification*, as well as the overall implementation infrastructure and mobile phone app.

1) Verification

The process of verifying a certificate is an on-demand action. A Verifier cannot validate a certificate unless requested. It requires a Holder to go to a Verifier for this purpose. A Verifier can be an employer or other individual or organisation to whom the Holder wants or needs to present the certificate. Fig. S1 shows the main data flows involved in Verification.

In Fig. S1, we see that once requested, at (A), the app reads the QR code from the Holder's phone. This QR code (which is generated from the data that itself is stored in the Solid Pod) has two components: the certificate and a URL pointing to the hash on the blockchain. At (B), the app extracts these components and at (C) locally generates a temporary hash of the certificate. Finally (D), the app fetches the hash stored on the blockchain and compares it with the local hash. The matching of the hashes indicates the validity and the authenticity of the certificate stored in the Solid Pod of the Holder. At the same time, the physical identity of the Holder can be confirmed by the Verifier via the Holder's photo ID which will already have been

'burned' into the mobile phone app certificate. The digital identity of the Holder can be confirmed by verifying the Verifiable Credential (embedded in the certificate) based on the relevant Holder DID.

2) The functional infrastructure

The components of our implementation communicate with each other via current Web standards — Hypertext Transfer Protocol Secure (HTTPS), RDF (primarily in the JSON-LD format), Verifiable Credentials, and Decentralized Identifiers — and via blockchain protocols (specifically, Ethereum protocols). The volumes of data and computational requirements are typically small and can be handled by a mobile device (full blockchain nodes are an exception, due to the potential size of the full chain data).

The main software functions required by the implementation are as follows:

Generate QR codes: Implemented using standard libraries to generate QR codes for identity and immunity certificates.

Generate hashes: Using standard libraries, certificates are transformed into a canonical RDF format before hashing, in order to ensure robust reproducibility of hashes, for verification.

Communicate with Blockchain: The Parity library is used to communicate with our Consortium blockchain. A light client library can handle read/write interactions with the blockchain without requiring a phone to maintain a full copy of the blockchain.

Communicate with Solid Pods: Communication with Solid takes place using the Solid REST API [18], to read and write personal data regarding the Holder to and from their Solid Pod with user permission.

Manage Issuer and Holder Credentials: Issuer and Holder credentials are stored in public/private key wallets containing DIDs. The authorization for an Issuer to create certificates can be represented as a Verifiable Credential issued by the relevant regulatory authority to the Issuer, which any participating party can verify. Currently we use Streetcred ID [19] to generate DIDs for the Issuers, Holders and Certificates.

Generate Verifiable Credentials: Certificates are created at issue time, and their contents asserted as the Claim elements in Verifiable Credentials to be stored in the Holder's Solid Pod, with metadata describing the relevant blockchain records forming the Proof. This provides a sharable data structure which permits anyone to check its authenticity.

3) The mobile phone app

Fig. S2 shows representative screen shots of the mobile phone app, which provides all the necessary UI elements for the Issuer, Holder and Verifier to perform their actions. At the time of writing, the main functionalities of the mobile phone app include the ability to scan and generate QR codes and generate hashes for text and images. For the QR code scan and generate functions to work, the mobile phone app is packed with necessary libraries to support QR code functions and only works on smartphones with built-in camera functionality. The mobile phone app also contains the hashing libraries. As the mobile phone app needs to communicate with a server, an active internet connection is necessary for HTTPS server calls.

For speed of implementation for the current prototype, a Node.js Express server does all the heavy lifting for the app, with the functionalities explained above. This is a temporary solution, however, given the urgency of the current situation.

C. Scenario variations

Throughout the paper we have focused on a scenario involving 'On-Site Test for Antibodies + Issuance of Digital Certificate Including Photo ID', but there are some key variations easily incorporated into our design, namely (i) 'Issuing Digital Certificate Without Photo ID', (ii) 'Issuing Paper Certificate', (iii) 'Off-Site Testing Via External Lab', and (iv) 'Vaccination + Certification', described in turn below.

1) Variation 1: Issuing Digital Certificate Without Photo ID

In our scenario in the main paper, Fig. 2, the Issuer (Pharmacy) needs to authenticate that the Holder is who they say they are, and thus requests that the Holder display both a physical ID, such as a Driving License or a Passport and also a QR code which is scanned by the Issuer using the Issuer's mobile phone app. At this point there is in fact a choice: the Issuer can either (a) tap to accept the ID, in which case the Holder's photo will be 'burned' into the upcoming steps so that at the final step of verification, there will be no need to display the same physical ID, or (b) leave the Holder to display the physical ID once again at verification time.

If path (b) is chosen, there are other implications. At Verification time, to avoid someone else impersonating the Holder, the Holder must present not only the certificate, but also some proof of identity. In this variation, the Verifier can confirm the identity of the Holder by visually inspecting a physical ID card, and separately scanning the Holder's presented QR code (without ID incorporated) to verify just the certificate.

2) Variation 2: Issuing Paper Certificate

At step 2 of our main scenario, the test certificate can in fact be provided purely on paper, which has a dual purpose for the Holder: (a) a fallback in case of mobile phone failure; (b) a 'tech-agnostic' option which enables us to provide certification in a more appropriate manner for cases of socio-economic deprivation. This alternative means that some of the advantage of digital certification will be missing, but the use of printed QR codes which include the image of the Holder are still a useful advance over plain paper certificates. It also provides an alternative for individuals with little access to technology, but for whom a paper-based QR code printout can serve as a 'good enough' and 'effectively tamper-proof' certificate.

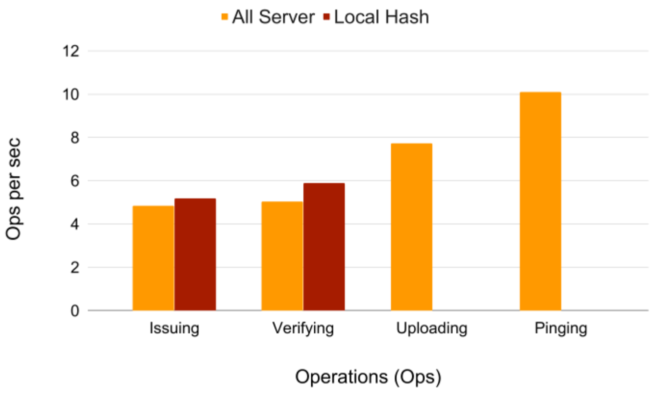

Fig. S3. Operations per second for Issuing (Server vs Local hashing), Verifying' (Server vs Local hashing), Uploading and (baseline) 'Pinging'.

3) Variation 3: Off-Site Testing Via External Lab

It is likely that in many cases, particularly where large volume or high-quality serology testing is required, the Holder's blood sample has to be sent to a separate lab for processing. In this variation, the Pharmacist can issue a certificate that is flagged as being in a 'pending' state. The lab technician will also have a login to the app, via an additional button on the login screen, and see the list of pending certificates waiting for processing and approval. Once the lab technician has the results for a blood sample, the technician has to scan the QR code attached to the sample (this incorporates the Holder's digital ID, but with no personal information exposed to the lab technician) and then tap a button to issue the certified results to the relevant Holder. At this point, the Holder receives a notification with details of the certified result.

Note that the steps in this variation are just like the steps in 'supply chain provenance' gaining increasing traction in the blockchain 'farm-to-fork' world, typified by the IBM Food Trust [20]. Such efforts are also gaining ground in the area of vaccine supply chain provenance [21]. At each step of the chain, each participant adds the information pertinent to their niche, and digitally signs, while cross-checking automatically for authenticity of provenance at earlier steps in the supply chain. For blood samples, both the issuer and lab technician would add serial numbers and details for the blood sample and containers, syringes as necessary, and respective registration numbers / IDs for their roles as pharmacist and lab technician. At Verifier stage, and even for the lab test manufacturer, similar procedures would be deployed so that the integrity of the whole testing life cycle was ensured.

4) Variation 4: Vaccination + Certification

Although the most forward-looking variation (because vaccine research, development, approval, and deployment may take the longest [2]), it fits very smoothly into our existing scenario life cycles. Essentially, the Issuer as described throughout the main section of the paper becomes the person administering the vaccination jab (as opposed to taking a blood sample), and certifying that this has happened in the same manner described for the antibody test certificate. The approach to 'supply chain provenance' discussed in the preceding paragraph also applies to this variation, because the Issuer will have to include details of the vaccination source and batch within the certificate.

III. Results

Fig. S3 shows the number of operations per second (Ops/sec) for Issuing, Verifying, Uploading, and Pinging, calculated from the slope of the 1-100 parallel operations timing described in the main paper. It demonstrates that while the current configuration is constant, our architecture can serve about five certificate issuances per second. For verifications, although we experimented with both local and server variants, in practice the hash will be generated locally (within the mobile phone app), giving us the ability to verify about six certificates per second with the existing infrastructure.

This observation shows us that the operations of Issuing and Verifying are twice as expensive as the simplest server ping. Except for some common infrastructure, the architecture is decentralized, i.e. one issuer issues (or verifier verifies) one certificate using one smartphone at a time even if we have hundreds of thousands of parallel requests. Even some commonly held infrastructure can be more distributed, such as the Solid pods. In this experiment, we used just one Solid cloud server for all requests, but in practice, users will have their Solid pod hosted on multiple servers or their own mobile phone. Therefore, if only those common and fixed infrastructures are scaled up, or load-balancing is applied to divert requests over multiple machines, performance time will significantly improve, with a concomitant speedup of Issuing and Verification not requiring architectural re-design.

IV. Discussion

A. More about rollout

The architecture presented in the main paper and Supplementary Material above is all built on standard library modules, and therefore joining a Consortium blockchain to help roll this out at scale is relatively straightforward, subject to suitable testing and deployment. The key hurdles are primarily Issuer credentials and the critical mass of the Consortium blockchain. In the case of Issuer credentials, we mentioned in section II.E.1 about Onboarding that we use two factor authentication for Issuers, and an API provided by the General Pharmaceutical Council to cross-check registration — this of course is subject to approval, and relevant discussions are already underway. As for the Consortium blockchain, a strong Consortium of industrial and academic partners needs to be established, after which addition of new members is just a matter of approval by the existing Consortium and the distribution of training and instruction materials. Alternatively, 'parallel' consortia can be created by cloning our approach.

Given related ongoing work [22] that we mentioned in the main paper, we are optimistic that critical mass can be achieved.

B. Ethical considerations

It should be clear from the previous sections that the concepts underlying Verifiable Credentials and the Decentralized Verification of Data with Confidentiality are diametrically opposed to any kind of central data storage or 'Big Brother'-style snooping and data collection, and indeed provide excellent and agreed standards for avoiding such snooping and data collection. To be clear, in the approach we advocate in this paper,

> *Personally identifiable information is stored entirely under the Holder's control (on a mobile phone, on the Holder's cloud provider of choice, or both), and additionally for later verification purposes in minimal (a few bytes) encoded form (hash) on a Consortium blockchain. Moreover, the app allows the user selectively to present only the specific test result, with no other personal information revealed.*

How is it possible that no personal information is stored in a database? What about the certificate itself? That's the beauty of Verifiable Credentials, Zero Knowledge Proofs and our approach of Minimum and Encoded Data Storage / User's Choice: taken together, this combined approach offers cryptographically signed, verifiable, un-tamperable proof that the certificate being shown was really granted by a known testing authority to the person in question, even without showing the name, address, phone number or even UK NHS number of the person holding it.

Everything in this app is decentralized. Anyone wishing to abandon involvement in this kind of certification can just delete the Verifiable Credentials stored on their Solid Pods. There will be no records whatsoever, as if they had never been on the system. Deleting data on the Solid Pods will also turn the hashes on the blockchain into 'orphans' (no data pointing to the hash), i.e. the hashes will become meaningless: it is not possible to recover the original data from a hash.

This almost-too-good-to-be-true approach does raise a fresh concern, raised briefly in the main paper: the same techniques we are advocating seem to open up what we call the *'Private Verifiable Credentials Paradox'*: your digital mobile phone app certificate is so much more private and tamper-proof than the old paper or database versions that it *could* (deliberately or accidentally), be weaponized *for discrimination against your fellow citizens*. In other words, a potential problem, according to critics, is not that the architecture is too weak, but that it is too strong.

Clearly, the more powerful methods of today and tomorrow have the potential to open up a Pandora's Box of Bad Use, if not by the modern democracies in which we may have grown up, then by *some* authority in another time or place - as the world has witnessed all too tragically in the past. We started this project with the noble aim of facilitating a way to get people back to work and heading towards recovery from the devastating impact of the Coronavirus Pandemic of 2019/2020. If COVID-19 antibodies can indeed be shown reliably to confer immunity, and the overwhelming support for the 'test-test-test' mantra of the World Health Organization continues to hold, then people *are* going to get tested, in overwhelming numbers, and certificates *are* going to be issued in one form or another.

But we are not adopting a 'give-up-and-accept-our-fate-in-the-hands-of-bad-actors' approach. Yes, a secure digital certificate could hypothetically be weaponized to a greater degree than a paper one, but the actual degree could be something of a mind-set illusion. *Any* certification method has such potential, and therefore, rather than casting the technology in terms of 'good vs evil' we think our approach is best considered as something that involves a trade-off between (a) the advantages of getting people back to work using good privacy-preserving fraud-prevention methods and (b) the disadvantages of discriminatory (mis)use of such methods. Our approach to this trade-off is strongly to nudge things towards (a), and therefore we propose the following concrete steps to achieve this:

- App usage should be strictly opt-in/optional: a paper certificate must always be allowed by default, just as with, say, train or airline tickets. This helps introduce the concept and technology in a gentle manner: people will ultimately decide what they prefer for themselves.
- Implementations must comply with UK NHS Information Governance (IG) guidelines [23], [24]. Compliance should in principle be straightforward, because (a) in our approach, personally identifiable information is stored entirely under the Holder's control, and additionally for later verification purposes in minimal hash-encoded form on a Consortium blockchain, and (b) the app allows the user selectively to present only the specific test result, with no other personal information revealed. Even so, the UK NHS IG documents provide a strong guiding framework for ensuring continuing compliance, particularly with respect to relevant EU GDPR requirements such as 'Right to erasure' and 'Right to data portability': our architecture by its very design avoids database storage of personally identifiable information, but oversight of possible misuse/abuse of this and related technologies needs to be maintained, as the next three bullet points suggest.
- COVID-19 Antibody Test Certificates should only be applied to workers in healthcare and other comparable key sectors, as defined by the appropriate UK Parliamentary process (for example, the list of key exceptions to mandatory business closure during the current pandemic was specified by the UK Ministry of Housing, Communities, and Local Government), with input from an Ethics Committee mentioned next.
- An Ethics Committee, comparable in scope and composition to the UK NHS Research Ethics Committees, should have oversight of actual deployment of the approach advocated herein.

- The approach should be reviewed on a 3-monthly basis.

In a timely and thoughtful analysis of the ethical complexities surrounding COVID-19 antibody test certificates, Persad and Emanuel [25] argue convincingly for the label 'immunity-based licenses' (rather than 'immunity passports') as a way to focus on the positive benefits granted to those who have been infected with COVID-19, without necessarily worsening the lives of those who have not been infected.

Ethical standards are challenging to uphold, but uphold them we must: we see a strong emphasis on ethics as the best way to negotiate a path towards a 'pandemic end game' in a manner acceptable to the widest possible audience.

V. Conclusions

Will such an app be suitable as part of a 'pandemic exit strategy' for helping get people back to work in key sectors? There are many issues to be addressed first, including the rigorous scrutiny and approval of antibody tests, likelihood and longevity of immunity, agreement concerning ethical oversight, and acceptance by the public. Our approach is intended to ensure that the procedures for creating tamper-proof, verifiable, privacy-preserving certificates are 'ready to go' while waiting for antibody/immunity tests to achieve the required state of robustness and acceptance. We believe that, just as with train e-tickets, end-users will 'vote with their feet' and deploy the app in large numbers once its benefits have been demonstrated. To take a stance against what we call the 'Pandora's Box of Bad Use', we proposed ethical guidelines at the end of the Discussion, which we believe are essential for the principled development and deployment of the prototype described in this paper.

References

[1] BBC News, "WHO head: 'Our key message is: test, test, test.'" [Online]. Available: https://www.bbc.co.uk/news/av/world-51916707/who-head-our-key-message-is-test-test-test [Accessed: Apr. 2, 2020].

[2] T. Thanh Le et al., "COVID-19 vaccine development landscape," *Nat Rev Drug Discov*. https://www.nature.com/articles/d41573-020-00073-5.

[3] A. Pethick, "Developing antibody tests for SARS-CoV-2," *Lancet*, vol. 395 (10230), pp. 1101–1102, Apr. 4, 2020. DOI: 10.1016/S0140-6736(20)30788-1

[4] BBC News, "'Immunity passports' could speed up return to work after Covid-19." [Online]. Available: https://www.theguardian.com/world/2020/mar/30/immunity-passports-could-speed-up-return-to-work-after-covid-19 [Accessed: Apr. 2, 2020].

[5] The Guardian, "No 10 seeks to end coronavirus lockdown with 'immunity passports.'" [Online]. Available: https://www.theguardian.com/politics/2020/apr/02/no-10-seeks-to-end-covid-19-lockdown-with-immunity-passports [Accessed: Apr. 3, 2020].

[6] CNBC, "WHO officials say it's unclear whether recovered coronavirus patients are immune to second infection." [Online.] https://www.cnbc.com/2020/04/13/who-officials-say-its-unclear-whether-recovered-coronavirus-patients-are-immune-to-second-infection.html [Accessed: Apr. 13, 2020].

[7] The New York Times, "After Recovery From the Coronavirus, Most People Carry Antibodies." [Online]. Available: https://www.nytimes.com/2020/05/07/health/coronavirus-antibody-prevalence.html. [Accessed: May 11, 2020].

[8] A. Wajnberg, M. Mansour, E. Leven *et al.* "Humoral immune response and prolonged PCR positivity in a cohort of 1343 SARS-CoV 2 patients in the New York City region." *medRxiv*. May 2020:2020.04.30.20085613. doi:10.1101/2020.04.30.20085613

[9] NPR Radio/Web. If You Get Coronavirus And Recover, Do You Develop Immunity? [Online]. Available: https://www.npr.org/sections/goatsandsoda/2020/03/20/819038431/do-you-get-immunity-after-recovering-from-a-case-of-coronavirus. [Accessed: Apr. 14, 2020] .

[10] D. Male, J. Golding, and M. Bootman, "How Does The Human Body Fight A Viral Infection?" Open University, OpenLearn Course Module, Milton Keynes, UK, 2020. [Online]. Available: https://www.open.edu/openlearn/science-maths-technology/biology/how-does-the-human-body-fight-viral-infection [Accessed: Apr. 7, 2020].

[11] "Global Progress on COVID-19 Serology-Based Testing." Johns Hopkins Bloombert School of Public Health, Center for Health Security." [Online]. Available: https://www.centerforhealthsecurity.org/resources/COVID-19/serology/Serology-based-tests-for-COVID-19.html. [Accessed: May 11, 2020].

[12] "About Sovrin.org." [Online]. Available: https://sovrin.org/ [Accessed: Apr. 14, 2020]

[13] Sovrin.org, "A Protocol and Token for Self-Sovereign Identity and Decentralized Trust." [Online]. Available: https://sovrin.org/wp-content/uploads/2018/03/Sovrin-Protocol-and-Token-White-Paper.pdf [Accessed: Apr. 14, 2020].

[14] R. Verborgh, "Paradigm shifts for the decentralized Web Online." [Online]. Available: https://ruben.verborgh.org/blog/2017/12/20/paradigm-shifts-for-the-decentralized-web/. [Accessed: Apr. 14, 2020].

[15] "Zero-knowledge proof." [Online]. Available: https://en.wikipedia.org/wiki/Zero-knowledge_proof [Accessed: Apr. 14, 2020].

[16] A. S. Delight, "Zero Knowledge Proof of Age Using Hash Chains." [Online]. Available: https://www.stratumn.com/thinking/zero-knowledge-proof-of-age-using-hash-chains/ [Accessed: Apr. 14, 2020].

[17] S. Angel and M. Walfish, "Verifiable Auctions for Online Ad Exchanges," *ACM SIGCOMM*, vol. 13. [Online]. Available: https://cs.nyu.edu/~mwalfish/papers/vex-sigcomm13.pdf [Accessed: Apr. 14, 2020].

[18] "Solid HTTPS REST API Spec." [Online]. Available: https://github.com/solid/solid-spec/blob/master/api-rest.md [Accessed: Apr. 14, 2020].

[19] "Getting Started with Streetcred ID." [Online]. Available: https://docs.streetcred.id/docs/getting-started [Accessed: Apr. 14, 2020].

[20] "IBM Food Trust - United Kingdom | IBM." [Online]. Available: https://www.ibm.com/uk-en/blockchain/solutions/food-trust. [Accessed: 13-May-2020].

[21] B. Yong, J. Shen, X. Liu, F. Li, H. Chen, and Q. Zhou, "An intelligent blockchain-based system for safe vaccine supply and supervision," Int. J. Inf. Manage., vol. 52, p. 102024, Jun. 2020. doi:10.1016/j.ijinfomgt.2019.10.009

[22] Coindesk. "COVID-19 'Immunity Passport' Unites 60 Firms on Self-Sovereign ID Project." [Online]. Available: https://www.coindesk.com/covid-19-immunity-passport-unites-60-firms-on-self-sovereign-id-project [Accessed: Apr. 19, 2020].

[23] "NHS Information Governance." [Online]. Available: https://www.england.nhs.uk/ig/about/ [Accessed: Apr. 9, 2020].

[24] "About the NHS IG Toolkit." [Online]. Available: https://www.igt.hscic.gov.uk/resources/About%20the%20IG%20Toolkit.pdf [Accessed: Apr. 9, 2020].

[25] G. Persad and E. Emanuel, "The Ethics of COVID-19 Immunity-Based Licenses ('Immunity Passports')." *JAMA*. May 6, 2020. [Online]. Available: https://dx.doi.org/10.1001/jama.2020.8102 [Accessed: May 20, 2020].
